# Caprine brucellosis: A historically neglected disease with significant impact on public health

**DOI:** 10.1371/journal.pntd.0005692

**Published:** 2017-08-17

**Authors:** Carlos A. Rossetti, Angela M. Arenas-Gamboa, Estefanía Maurizio

**Affiliations:** 1 Instituto de Patobiología, CICVyA-CNIA, INTA. Nicolás Repetto y de Los Reseros s/n, Hurlingham, Buenos Aires, Argentina; 2 Department of Veterinary Pathobiology, College of Veterinary Medicine & Biomedical Sciences, Texas A&M University, College Station, Texas, United States of America; King Saud University College of Medicine, SAUDI ARABIA

## Abstract

Caprine brucellosis is a chronic infectious disease caused by the gram-negative cocci-bacillus *Brucella melitensis*. Middle- to late-term abortion, stillbirths, and the delivery of weak offspring are the characteristic clinical signs of the disease that is associated with an extensive negative impact in a flock’s productivity. *B*. *melitensis* is also the most virulent *Brucella* species for humans, responsible for a severely debilitating and disabling illness that results in high morbidity with intermittent fever, chills, sweats, weakness, myalgia, abortion, osteoarticular complications, endocarditis, depression, anorexia, and low mortality. Historical observations indicate that goats have been the hosts of *B*. *melitensis* for centuries; but around 1905, the Greek physician Themistokles Zammit was able to build the epidemiological link between “Malta fever” and the consumption of goat milk. While the disease has been successfully managed in most industrialized countries, it remains a significant burden on goat and human health in the Mediterranean region, the Middle East, Central and Southeast Asia (including India and China), sub-Saharan Africa, and certain areas in Latin America, where approximately 3.5 billion people live at risk. In this review, we describe a historical evolution of the disease, highlight the current worldwide distribution, and estimate (by simple formula) the approximate costs of brucellosis outbreaks to meat- and milk-producing farms and the economic losses associated with the disease in humans. Successful control leading to eradication of caprine brucellosis in the developing world will require a coordinated Global One Health approach involving active involvement of human and animal health efforts to enhance public health and improve livestock productivity.

## Author summary

Human brucellosis is an ancient disease that has had different names throughout time based on the main clinical symptom (fever) and the geographical location: Malta fever, Mediterranean fever, Undulant fever, Gibraltar fever, Rock fever, and Neapolitan fever, among others. Retrospective studies have demonstrated that goats have been the hosts of *B*. *melitensis* for centuries, with evidence of its zoonotic potential early in evolution. Since domestication of goats (and also sheep), the incidence of human brucellosis has been on the rise, becoming endemic in resource-limited settings. Today, millions of goats and approximately half of the human population worldwide live at risk. For that reason, more effective prevention and control measures, such as affordable vaccines, more sensitive and specific diagnostic techniques, and the control livestock movement, among others, are desperately needed to control and eradicate brucellosis in goats and to prevent human brucellosis.

## Introduction

*Brucella melitensis* is the etiological agent of caprine brucellosis, an infectious zoonotic disease with significant economic impact on both the livestock industry and public health. Worldwide, there are approximately 1 billion goats, with an increase of the population by more than 20% in the last 10 years. Approximately 90% of goats are located in the developing world, where they are considered one of the most important sources of protein for humans [1]. Caprine brucellosis has been controlled in most industrialized countries; however, this disease remains endemic in resource-limited settings, where small ruminants are the major livestock species and the main economical livelihood, such as the Mediterranean region, the Middle East, Central Asia, sub-Saharan Africa, and parts of Latin America [2]. Among the different *Brucella* spp. capable of causing disease in humans (*B*. *abortus*, *B*. *melitensis*, *B*. *canis* and *B*. *suis*), *B*. *melitensis* is the most virulent [3]. Human brucellosis has had different names throughout time based on the main clinical symptom (fever) and the geographical location: Malta fever, Mediterranean fever, Undulant fever, Gibraltar fever, Rock fever, and Neapolitan fever, among others [4]. Brucellosis is considered a severely debilitating and disabling illness that results in high morbidity with intermittent fever, chills, sweats, weakness, myalgia, abortion, osteoarticular complications, endocarditis, depression anorexia, and low mortality. Due to causing a protracted, incapacitating disease with minimal mortality, the low infectivity dose required to cause infection (10–100 colony-forming units), and the potential for aerosol dissemination, *B*. *melitensis* was considered a potential bioterrorist agent early in the 20th century [5]. Gradually, biological warfare moved on, and *Brucella*’s perceived status as a potential agent of bioterrorism declined, until the World Trade Center attack in 2001 brought bioterrorism back to the public’s attention. Nowadays, *B*. *melitensis* possession and use is still strictly regulated in the United States of America, Canada, and some European countries. Conversely, more than half a million new brucellosis cases per year occur naturally in the populations of developing areas of the world, a number which is thought to be severely underestimated [6].

## Methods

A MEDLINE (via Pubmed) and SCOPUS online databases search for articles with “*Brucella melitensis*” or “brucellosis” and “goats” or “small ruminants” as keywords with no date limit and published up to December 31st, 2016, was performed. An additional internet search was done in Google without language restriction, using those terms and including country names. Early reports were obtained from original printouts from the reference list of selected articles and printed books.

### Historical evolution

Despite the first scientific evidence that goats were the reservoir host of *B*. *melitensis* in 1905, several observations would indicate that goats have been the host of *B*. *melitensis* for centuries [7]. Phylogenetic studies suggest that brucellosis in goats emerged in the past 86,000 to 296,000 years through contact with infected sheep [8]. Interestingly, to support this observation, a recent study found lesions in vertebral bodies of an *Australopithecus africanus* (who lived 2.5 million years ago) consistent with brucellosis, where the source of infection could be the consumption of infected tissues from wild animals [9]. Subsequently, the closer association of humans with goats (and also sheep) due to domestication around 10,000 years ago [10] favored an increase in the incidence of human brucellosis. As essential resources for human survival, goat and sheep herds moved along with human communities from the Fertile Crescent in Southwestern Asia to lands around the Mediterranean Sea [11], where Phoenician traders might have contributed to the spread of *B*. *melitensis* infection throughout the Mediterranean littoral and islands during the first millennium B.C. [12]; it was then introduced to the Americas around the 16th century by Spanish and Portuguese conquerors [11,13].

The first written evidence of goat brucellosis could be inferred from the first description of 2 human cases of brucellosis. In the 4th century B.C., in his *Epidemics* book, Hippocrates II described 2 cases of a 120-day fever in people living in the Mediterranean littoral, most likely associated with the consumption of raw milk or derivatives of *B*. *melitensis*-infected sheep or goats [14]. Another testimony of the ancient presence of caprine brucellosis comes from preserved evidence from the volcanic eruption of Mount Vesuvius in Italy on August 25th in the year 79 A.D. Scanning electron microscopy examination of remnants of carbonized cheeses revealed cocci-like forms consistent with *B*. *melitensis*, while an anthropological examination of human skeletal remains from that incident revealed an arthritic condition consistent with brucellosis [7]. References to and vivid descriptions of clinical cases compatible with human brucellosis were continuously reported in histories of military campaigns and hospital reports [15]. However, the identification of the etiological agent, the reservoir, and the epidemiology of the disease was not unraveled until the second half of the 19th century, when the British government decided to find a solution for their troops stationed on the island of Malta that annually suffered substantial losses caused by the so-called “Malta fever.” In 1859, British Army surgeon Jeffery Marston contracted what he called “Mediterranean remittent fever” [16]. After recovering, he described his own case in great detail, being the first author to clinically and pathologically differentiate human brucellosis from typhus, typhoid, and other prevalent fevers [15]. In 1884, the Australian-born British physician David Bruce was deployed to Malta to investigate the cause of “Malta fever” (later called brucellosis in his honor). Late in 1886, using a microscope, he observed a great number of micrococci in a fresh preparation of the splenic pulp of soldiers who had died from the disease [17]. One year later, Sir Bruce isolated the causative agent of “Malta fever” (which he called *Micrococcus melitensis* and then renamed *Brucella melitensis*) from samples of spleens of 4 patients inoculated into Koch’s nutrient agar and was able to reproduce the disease in monkeys following Koch’s postulates [18]. A few years later, Professor Almroth Edward Wright developed a serum agglutination test and demonstrated the presence of specific agglutinins in the blood of infected patients, which helped differentiate those who suffered “brucellosis” from those with typhoid (cholera) or malarial fever [19]. The use of this serological test in goats provided the first insights into the epidemiology of the disease. In 1904, a Public Health Officer of Malta discovered that the blood of goats that supplied milk to people that had contracted “Malta fever” had agglutinins against *M*. *melitensis*, and a posterior survey indicated that around 50% of Malta’s goats’ blood reacted to this microorganism. This observation suggested that goats were susceptible to natural infection with *M*. *melitensis*. Based on all knowledge available on brucellosis, the Greek physician Themistokles Zammit hypothesized that goats were susceptible to Malta fever and that the disease spread from goats to human. To test his hypothesis, Zammit fed seronegative, healthy goats with agar cultures of *M*. *melitensis* mixed into their food. Goats became seropositive to *M*. *melitensis* after 20 days or more, and *Brucella* was isolated from the milk, blood, and urine of infected animals without any clinical manifestation of the disease [20]. This simple assay demonstrated that goat milk was the disseminating vehicle of the bacteria, rather than an insect vector, and helped to build the epidemiological link of “Malta fever” to the consumption of this product. This observation was further confirmed after its ban from the diet of the Malta garrison significantly reduced the incidence of brucellosis in the army and naval forces compared to the general population of Malta that continued to consume contaminated dairy products. Later on, in 1918, Alice Evans demonstrated the similar characteristics between the *M*. *melitensis* and the etiological agent of bovine epizootic abortion, the “abortus bacillus” (now *Brucella abortus*), isolated by a Danish veterinarian Bernhard Bang in 1896, and based on that, both agents were included under the same bacterial genus (*Brucella*) in honor of David Bruce, in 1920. Major events of caprine brucellosis and its relationship with public health throughout history are summarized in **Table 1**.

**Table 1:** Dates and major events associated with caprine brucellosis and its relationship with public health throughout history.

When	Who	What	Reference
9th to 7th centuries B.C.	Neolithic population	Initial domestication of goats in the Fertile Crescent (Asia), evidence for the milking of goats	[11]
400 B.C.	Hippocrates II	First clinical description of human brucellosis	[14]
79	N/A	Human skeletons with arthritic condition consistent with brucellosis	[7]
1859	Jeffery Marston	First author to differentiate human brucellosis from other prevalent fevers	[16]
1887	David Bruce	Isolated the causative agent of “Malta fever” (*Micrococcus melitensis*) for the first time and reproduced the disease in monkeys	[18]
1904	Anonymous Public Health Officer of Malta	Found agglutinins against *M*. *melitensis* in Maltese goats’ blood	[20]
1905	Themistokles Zammit	Identified Maltese goats’ milk as the source of infection for human brucellosis	[20]
1957	Elberg and Faunce	*B*. *melitensis* strain Rev.1 was successfully evaluated as immunogen against caprine brucellosis	[21]

### World distribution

Caprine brucellosis refers to goat herds infected with *B*. *melitensis*. Goats can be susceptible to *B*. *abortus* infection [22,23,24] under particular epidemiological situations (for instance, when goats live in close contact with *B*. *abortus*-infected cattle); however, these individuals don’t sustain the infection in the herd. Similarly, *B*. *suis* isolations from goats have seldom been reported, but in recent times, they have not been further documented [25]. *B*. *melitensis* comprises 3 biovars (1–3), distinguished solely by their immunochemical reactions with monospecific anti-lipopolysaccharide (LPS) A- and M-determinant sera [26,27]. Available information indicates that most infections are caused by biovars 1 and 3 [28], both of which seem to have similar virulence for goats and humans.

**Fig 1:**
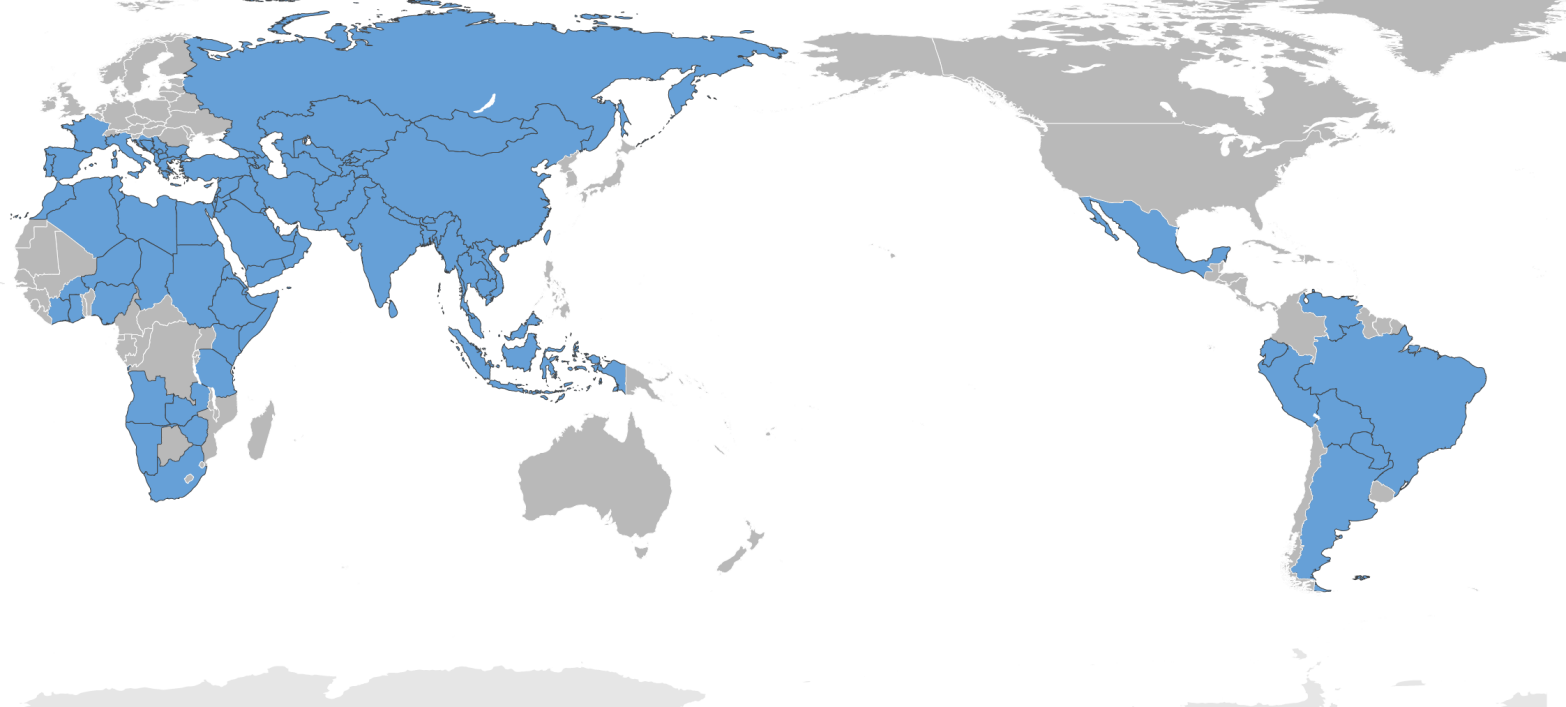
Caprine brucellosis worldwide distribution. Countries colored in blue indicate those countries where goats, human, cattle, or sheep brucellosis due to *B*. *melitensis* infection have been reported in recent years (2005–present). Countries in grey indicate that the disease is not present or that the status of the disease is unknown. The list of the countries is detailed in Table 2. The countries were colored using Adobe Illustrator CS6 software. *Base map credit*: *https://en.wikipedia.org/wiki/World_map#/media/File:BlankMap-World-162E-flat.svg*.

Prevalence of caprine brucellosis around the world has been reported and referenced by others [28,29,30,31,32]. The disease is present in 5 out of the 7 continents (South and North America, Europe, Asia, and Africa). Despite being under control in most industrialized countries, it remains a major problem in the Mediterranean region, the Middle East, Central and Southeast Asia, sub-Saharan Africa, and parts of Latin America (**Fig 1**).

As expected, prevalence of human brucellosis is also high in those regions where goat brucellosis occurs [6]. The disease has been historically underreported, probably because low-income countries prioritize other diseases or lack facilities, human capabilities, and/or specific tests that would otherwise underpin diagnoses and research. Over the last 15 years, the infection has re-emerged, in particular in Eastern Europe, the Balkans, and Eurasia [2]. **Table 2** shows those countries where caprine brucellosis (i.e., presence of anti-*Brucella* antibodies, *B*. *melitensis* isolation, or *Brucella* DNA detection from goat samples) or brucellosis in humans, sheep, or cattle due to *B*. *melitensis* infection have been reported in recent years (2005–present). Historically, *B*. *melitensis* biovar 1 is predominant in Latin America [28,33], while biovar 2 is predominant in the Middle East together with biovar 3, which is also more common in European and African Mediterranean countries, Eurasia, and China [2,28,31,34,35]; biovars 1 and 3 seem to be equally present in India [36,37]. Unfortunately, there are few studies addressing the characterization of isolates from sub-Saharan countries.

**Table 2:** Countries where caprine brucellosis or brucellosis in humans, sheep, or cattle due to *B*. *melitensis* infection have been reported in recent years (2005–present).

Country	Level of detection	Reference
**America**		
Argentina	Anti-*Brucella* Ab in goats; isolation of *B*. *melitensi*s bv1 from goats	[38,39,40]
Bolivia and Brazil	Mentioned as countries with endemic caprine brucellosis	[28]
Ecuador	Anti-*Brucella* Ab in goats; *Brucella* spp. DNA detection in goat milk and LN	[41]
Mexico	Anti-*Brucella* Ab in goats; isolation of *B*. *melitensis* from goats	[42,43,44]
Paraguay	Human case of brucellosis by contact with infected goats: clinical and serological evidence	[45]
Peru	Anti-*Brucella* Ab in goats	[46,47]
Venezuela	Anti-*Brucella* Ab in goats	[48]
**Europe**		
Bosnia-Herzegovina	Anti-*Brucella* Ab in goats and human	[49,50]
Bulgaria	Anti-*Brucella* Ab in goats and human	[51,52]
Croatia	Anti-*Brucella* Ab and isolation of *B*. *melitensis* bv3 from goats	[53]
France	Anti-*Brucella* Ab in goats; isolation of *B*. *melitensis* bv3 from wild goats	[54]
Greece	Anti-*Brucella* Ab in goats and human; isolation of *B*. *melitensis* bv3 from human	[55]
Italy	Isolation of *B*. *melitensis* bv1 and bv3 from goats	[56,57]
Macedonia	Anti-*Brucella* Ab in goats	[58,59]
Portugal	Anti-*Brucella* Ab in goats	[60]
Serbia	Anti-*Brucella* Ab in goats and human	[61]
Spain	Anti-*Brucella* Ab in goats	[62]
Andorra, Albania, Montenegro, and Cyprus	Mentioned as countries with endemic caprine brucellosis	[28]
**Asia**		
Afghanistan	Anti-*Brucella* Ab in goats and sheep	[63]
Armenia	Anti-*Brucella* Ab in goats	[64]
Azerbaijan	Anti-*Brucella* Ab in goats and sheep	[65]
Bangladesh	Anti-*Brucella* Ab in goats and sheep	[66]
China	Isolation of *B*. *melitensis*; Anti-*Brucella* Ab in human	[35,67]
Georgia	Anti-*Brucella* Ab in goats; isolation of *B*. *melitensi*s from goats, sheep and cattle	[68,69]
India	Anti-*Brucella* Ab in goats and sheep; isolation of *B*. *melitensi*s bv3 from goats	[37,70]
Iran	Anti-*Brucella* Ab in goats and sheep; isolation of *B*. *melitensis* bv1 and 2 from sheep and human	[71,72,73]
Iraq	Anti-*Brucella* Ab in sheep and isolation of *B*. *melitensi*s from human	[74,75]
Israel	Anti-*Brucella* Ab in goats and human; isolation of *B*. *melitensi*s bv1 and 2 from goats	[76]
Jordan	Anti-*Brucella* Ab in goats and isolation of *B*. *melitensi*s bv3 from goat fetus and vaginal fluid	[77,78]
Kazakhstan	Anti-*Brucella* Ab in goats and sheep; isolation of *B*. *melitensi*s from blood of sheep	[79,80,81]
Kuwait	Isolation of *B*. *melitensis* bv2 from aborted bovine fetus	[82]
Kyrgyz Republic and Uzbekistan	Anti-*Brucella* Ab in goats and sheep	[80]
Lao	Anti-*Brucella* Ab in goats	[83]
Malaysia	Isolation of *B*. *melitensis* from vaginal fluid and spleen of goats	[84]
Mongolia	Anti-*Brucella* Ab in goats and sheep	[85]
Nepal	Anti-*Brucella* Ab in goats	[86,87]
Pakistan	Anti-*Brucella* Ab in goats	[88,89]
Palestine	Anti-*Brucella* Ab in goats; DNA detection and isolation of *B*. *melitensi*s bv3 from goat milk and fetus	[90,91]
Russia	Anti-*Brucella* Ab in goats and sheep; isolation of *B*. *melitensi*s bv3 from goats	[92]
Saudi Arabia	Anti-*Brucella* Ab in goats and sheep	[93]
Syria	Anti-*Brucella* Ab and isolation of *Brucella* spp. in sheep	[94]
Tajikistan	Anti-*Brucella* Ab in goats and sheep	[95]
Thailand	Anti-*Brucella* Ab in goats and sheep	[96,97]
Turkey	Anti-*Brucella* Ab in goats and human; isolation of *B*. *melitensi*s from goats	[98,99]
UAE	Anti-*Brucella* Ab in goats, sheep and camels	[100,101]
Lebanon, Bahrein, Qatar, Oman, Yemen, Turkmenistan, Myanmar, Cambodia, Vietnam, Brunei, Indonesia, Sri Lanka, and Singapore	Mentioned as countries with presence of caprine brucellosis	[28,29,31,100,102,103]
**Africa**		
Algeria	Anti-*Brucella* Ab in goats and human; isolation of *B*. *melitensis* bv3 from goats	[104,105]
Egypt	Anti-*Brucella* Ab in goats and sheep; isolation of *B*. *melitensis* bv3 from goats	[106,107]
Ethiopia	Anti-*Brucella* Ab in goats and sheep and isolation of *B*. *melitensis*	[108,109]
Ghana	Anti-*Brucella* Ab in goats and sheep	[110]
Kenya	Anti-*Brucella* Ab in goats and human	[111]
Libya	Anti-*Brucella* Ab in goats and human	[112]
Morocco	Anti-*Brucella* Ab in goats	[113]
Namibia	Anti-*Brucella* Ab in goats	[114]
Niger	Anti-*Brucella* Ab in goats and sheep	[115]
Nigeria	Anti-*Brucella* Ab in goats	[116,117]
Somalia	Anti-*Brucella* Ab in goats and sheep	[118]
South Africa	Anti-*Brucella* Ab in goats; isolation of *B*. *melitensis* bv1 from human	[119]
Sudan	Anti-*Brucella* Ab in goats and sheep; isolation of *B*. *melitensis* bv1 from ram	[120,121,122]
Tanzania	Anti-*Brucella* Ab in goats	[123,124]
Uganda	Anti-*Brucella* Ab in goats and human	[125,126]
Zimbabwe	Isolation of B. melitensis bv1 from goat	[127]
Tunisia, Chad, Cape Verde, Ivory Coast Burkina Faso, Eritrea, Djibouti, Angola, Zambia, Zimbabwe, and Swaziland	Mentioned as countries with presence of caprine brucellosis	[28,30,32,128]

**Abbreviations:** Ab, antibodies; bv, biovar

In the Americas, *Brucella melitensis* was most likely introduced around the 16th century via the infected goats and sheep of Spanish and Portuguese conquerors [11]. Today, *B*. *melitensis* is endemic in some regions of Mexico, Peru, and Argentina [28] and has also been reported in Ecuador and Venezuela [41,48]. Caprine brucellosis is apparently absent in Central America, Bolivia, Paraguay, and Brazil, although this epidemiological situation is not confirmed [129]. Goat herds from the USA, Canada, Colombia, Chile, and Uruguay are free from *B*. *melitensis* infection, and human cases in these countries are clearly associated with international travelers or infected food imported from endemic regions [6].

Despite intense joint efforts to eliminate *B*. *melitensis* from goat flocks in Europe, the disease still occurs in Portugal, Spain, France, Italy, the Balkans, Bulgaria, and Greece. Northern and Central European countries like the United Kingdom, Belgium, the Netherlands, Denmark, Germany, Austria, Switzerland, the Czech Republic, Hungary, Poland, Romania, Sweden, Norway, and Finland, among others, are officially free of the disease [129].

In Asia, brucellosis is broadly distributed. Except for Japan and the Republic of Korea (South Korea), where the disease has never been reported, caprine brucellosis is officially recognized in several countries on the continent, such as Turkey, Israel, Jordan, Iraq, Iran, Armenia, Georgia, Afghanistan, Russia, and Mongolia, among others (see references in **Table 2**), and is also known to be endemic in countries like Syria, Lebanon, India, China, Indonesia, Myanmar, etc., where no public information is available or the distribution of the information is restricted [28,29,102,129].

In Africa, caprine brucellosis is endemic in Mediterranean countries like Morocco, Algeria, Tunisia, Libya, and Egypt, and also in those countries located in the eastern part of the continent, such as Sudan, Eritrea, Ethiopia, Somalia, Kenya, Uganda, and Tanzania (see **Table 2** for references). Unfortunately, there is no information available from Central and West African countries like Chad, Congo, Angola, Zambia, Cameroon, Mali, Cote d’ Ivoire, Guinea, and Senegal, among others, where goats are abundant [130]. Altogether, the information above indicates that the knowledge regarding distribution of caprine brucellosis as well as the presence of *B*. *melitensis* around the world is sparse, especially in some areas of the Americas, Asia and Africa. The lack of useful epidemiological data must stimulate official veterinary services and public health officers to collect and share data for designing control and eradication plans.

### Economic impact: Direct and indirect cost in human health and goat production

Since brucellosis is considered a neglected disease that significantly affects countries where resources are limited, there are only a few studies that measure the economic impact of brucellosis in small ruminants. Sulima and Venkataraman (2010) and Singh et al. (2015) estimated the annual loss in India at Rs. 2,121 per goat (around US$39) and at US$71 million total, respectively [131,132]. Brisibe et al. (1996) calculated a loss of US$3.2 million per annum in 2 states of Nigeria [133], and more recently, Bamaiyi et al. (2015) reported the annual economic impact in Malaysia due to caprine brucellosis at almost US$2.6 million [134]. However, every publication utilizes different criteria, which makes comparisons difficult. A simple analysis of economic impact of caprine brucellosis on meat goat farmers can be calculated by taking into consideration the culling of animals serologically positive for *Brucella*, the abortions and stillbirths, the cost of veterinary services, and miscellaneous factors arising from brucellosis on farms. The economic loss for culling 1 reactor animal is equal to the market price of a healthy goat purchased for its replacement, minus the amount perceived for selling the positive reactor to a slaughterhouse. An abortion or stillbirth must be considered as loss of profit and its value calculated as the market value of a 6-month-old kid (which weighs around 10 kg). Veterinary services include visits to the farm, professional assistance, and serological surveys, while miscellaneous factors—such as man hours for taking care of ill flocks, reduced weight gain, the increased morbidity of weak offspring and low birth weight kids, any interest paid on money borrowed from banks, etc.—are variable and, therefore, difficult to predict and calculate. Based on these premises, it is possible to roughly estimate the economic impact of a brucellosis outbreak in a meat goat herd. For instance, in Argentina, the impact of a brucellosis outbreak in a flock of 100 goats, in which 25 does abort and 10 others become serologically positive, would be:

A) Replacement of animals (healthy female Creole crossbreed 1-year-old): US$50 each × 35 does = US$1,750
B) Culled animals: US$20 × 35 goats = US$700
C) Six-month-old kids: US$30 each x 25 goats = US$750
D) Veterinary assistance: US$200 (every farm visit)
E) Individual serological tests: US$4 (includes disposables and 1 serological test [Rose Bengal or Fluorescent Polarization assay]) × 100 animals × 2 (2 rounds of survey minimum) = US$800

$$\begin{array}{l} \mathrm{Economic}\;\mathrm{impact}=\mathrm{US}\$\;[(A-B)+C+D+E]\\ \;=\mathrm{US}\$\;[(1,750-700)+750+200+800]\\ \;=\mathrm{US}\$2,800 \end{array}$$

There are some differences if the analysis is done for a dairy goat farm. For instance, the market price of healthy milking goats (Anglo Nubian, Saanen, Toggenburg) is higher than for meat goats, and the loss of milk yield due to culled does has to be taken into account as well. Thus, a conservative impact of a brucellosis outbreak in a herd of 100 milk goats, in which 25 does abort and 10 others become serologically positive, would be:

A) Replacement of animals (healthy 1-year-old female): US$100 × 35 does = US$3,500
B) Culled animals: US$20 × 35 goats = US$700
C) Lower milk yield: US$4/L of milk farm sale × 500 L/lactation period × 35 culled animals = US$70,000
D) Veterinary assistance: US$200 (every farm visit) × 3 = US$600
E) Individual serological tests: US$4 (include disposables and 1 serological test [Rose Bengal or Fluorescent Polarization assay]) × 100 animals × 3 (3 rounds of survey minimum) = US$1,200

$$\begin{array}{l} \mathrm{Economic}\;\mathrm{impact}=\mathrm{US}\$\;[(A-B)+C+D+E]\\ \;=\mathrm{US}\$\;[(3,500-700)+70,000+600+1,200]\\ \;=\mathrm{US}\$74,600 \end{array}$$

The estimated cost will vary with the location, production system, facilities, and miscellaneous factors included. The calculations need to include additional losses due to the socioeconomic and indirect health effects of the disease in humans. Still today, human brucellosis is an underreported disease, often mistaken for malaria and typhoid fever (Halliday et al., 2015). WHO estimates around 500,000 new cases reported and an equal number of nonreported cases of human brucellosis each year, a high proportion of them caused by *B*. *melitensis*. Vulnerable populations include not only dairy goat and sheep farmers, small ruminant ranchers (especially in marginalized goat-keeping communities), and veterinarians and abattoir workers, but also lab personnel and consumers of unpasteurized dairy products.

Economic losses caused by the disease in humans arise from the cost of hospital treatment, medicines, patient out-of-pocket treatment expenses, and loss of work days and income due to illness. In Spain, losses by brucellosis were estimated at 790,000 pesetas per patient (US$5,030) [135], while in New Zealand, the approximated cost per patient was NZ$3,200 (US$2,250) [136]. In Africa, the cost of treating a patient ranges from 9 EUR in Tanzania to 200 EUR in Morocco and as much as 650 EUR in Algeria [128]. In Argentina, the annual treatment cost of brucellosis was estimated to be US$4,000 [137].

Traditional recommended antibiotic treatment for human brucellosis consists of 100 mg of doxycycline twice a day per os for 45 days combined with 1 g of streptomycin daily intramuscular (IM), 15 to 21 days; gentamycin 5 mg/kg/day (300–350 mg) IM, 7 to 10 days; or, alternatively, rifampicin 15mg/kg/day (600–900 mg) per os for 45 days [3]. Today, in Argentina, the cost for antibiotic treatment for a single patient is approximately US$200–US$300. This value does not include lost profit, laboratory analysis and X-ray images, medical expenses, and other miscellaneous expenses. Considering a complete health treatment, the cost for every brucellosis-infected person is up to US$1,000.

### Conclusions and future approaches

Brucellosis in small ruminants remains a significant burden on animal and human health in the developing world. Small ruminant owners and governments where brucellosis is endemic do not usually have enough economic resources nor technical expertise or facilities to afford control or eradication campaigns. On the other hand, *B*. *melitensis* is the *Brucella* species with the highest zoonotic potential, and in humans, it frequently presents nonspecific clinical symptoms similar to other infectious diseases that are also present in brucellosis-endemic areas [138]. Thus, the challenge of clinical–differential diagnosis adds to the inequality of accessible healthcare facilities in most developing countries. These cumulative issues contribute to brucellosis remaining endemic and neglected in resource-limited regions of the world.

The future major challenges include developing a more effective and affordable DIVA (differentiating infected from vaccinated animals) vaccine against small ruminant brucellosis for massive protection in endemic areas. Undoubtedly, this goal must be accompanied by an integrated control strategy with a massive vaccination campaign, strict epidemiological surveillance, and controlled movement of animals. Meanwhile, current efforts must focus on controlling new outbreaks using available tools to prevent *B*. *melitensis* transmission to humans.

**Key learning points**

- *Brucella melitensis* is the etiological agent of caprine brucellosis and the species of the *Brucella* genus with the highest zoonotic potential.
- Lesions in vertebral bodies consistent with brucellosis were found in an *Australopithecus africanus*, an ancient human who lived 2.5 million years ago.
- The disease remains a significant burden on goats and human health in the Mediterranean region, the Middle East, Central and Southeast Asia (including India and China), sub-Saharan Africa, and parts of Latin America.
- *B*. *melitensis* comprises 3 biovars (1–3): biovar 1 is predominant in Latin America, biovar 2 is predominant in the Middle East, and biovar 3 is also present in Middle East and also in European and African Mediterranean countries, Eurasia, and China. Biovars 1 and 3 seem to be equally present in India.
- The economic impact of a brucellosis outbreak is higher in milk- than in meat-producing farms.

**Top five papers**

1. Benkirane A (2006) Ovine and caprine brucellosis: World distribution and control / eradication strategies in West Asia / North Africa region. Small Ruminant Research 62: 19–25.
2. Bruce D (1893) Sur une nouvele forme de fievre. Annales de l'Institut Pasteur 7: 289–304.
3. FAO. *Brucella melitensis* en Eurasia and the Middle East. In: FAO, editor; 2010; Rome, Italy. pp. 57 pp.
4. Foster JT, Beckstrom-Sternberg SM, Pearson T, Beckstrom-Sternberg JS, Chain PS, et al. (2009) Whole-genome-based phylogeny and divergence of the genus Brucella. J Bacteriol 191: 2864–2870.
5. Pappas G, Papadimitriou P, Akritidis N, Christou L, Tsianos EV (2006) The new global map of human brucellosis. Lancet Infect Dis 6: 91–99.
